# FibroVit—Vision transformer-based framework for detection and classification of pulmonary fibrosis from chest CT images

**DOI:** 10.3389/fmed.2023.1282200

**Published:** 2023-11-08

**Authors:** Muhammad Waseem Sabir, Muhammad Farhan, Nabil Sharaf Almalki, Mrim M. Alnfiai, Gabriel Avelino Sampedro

**Affiliations:** ^1^Department of Computer Science, COMSATS University Islamabad, Sahiwal, Pakistan; ^2^Department of Special Education, College of Education, King Saud University, Riyadh, Saudi Arabia; ^3^Department of Information Technology, College of Computers and Information Technology, Taif University, Taif, Saudi Arabia; ^4^Faculty of Information and Communication Studies, University of the Philippines Open University, Los Baños, Philippines; ^5^Center for Computational Imaging and Visual Innovations, De La Salle University, Manila, Philippines

**Keywords:** pulmonary fibrosis, vision transformer, ViT, classification, computed tomography (CT), deep learning, detection

## Abstract

Pulmonary Fibrosis (PF) is an immedicable respiratory condition distinguished by permanent fibrotic alterations in the pulmonary tissue for which there is no cure. Hence, it is crucial to diagnose PF swiftly and precisely. The existing research on deep learning-based pulmonary fibrosis detection methods has limitations, including dataset sample sizes and a lack of standardization in data preprocessing and evaluation metrics. This study presents a comparative analysis of four vision transformers regarding their efficacy in accurately detecting and classifying patients with Pulmonary Fibrosis and their ability to localize abnormalities within Images obtained from Computerized Tomography (CT) scans. The dataset consisted of 13,486 samples selected out of 24647 from the Pulmonary Fibrosis dataset, which included both PF-positive CT and normal images that underwent preprocessing. The preprocessed images were divided into three sets: the training set, which accounted for 80% of the total pictures; the validation set, which comprised 10%; and the test set, which also consisted of 10%. The vision transformer models, including ViT, MobileViT2, ViTMSN, and BEiT were subjected to training and validation procedures, during which hyperparameters like the learning rate and batch size were fine-tuned. The overall performance of the optimized architectures has been assessed using various performance metrics to showcase the consistent performance of the fine-tuned model. Regarding performance, ViT has shown superior performance in validation and testing accuracy and loss minimization, specifically for CT images when trained at a single epoch with a tuned learning rate of 0.0001. The results were as follows: validation accuracy of 99.85%, testing accuracy of 100%, training loss of 0.0075, and validation loss of 0.0047. The experimental evaluation of the independently collected data gives empirical evidence that the optimized Vision Transformer (ViT) architecture exhibited superior performance compared to all other optimized architectures. It achieved a flawless score of 1.0 in various standard performance metrics, including Sensitivity, Specificity, Accuracy, F1-score, Precision, Recall, Mathew Correlation Coefficient (MCC), Precision-Recall Area under the Curve (AUC PR), Receiver Operating Characteristic and Area Under the Curve (ROC-AUC). Therefore, the optimized Vision Transformer (ViT) functions as a reliable diagnostic tool for the automated categorization of individuals with pulmonary fibrosis (PF) using chest computed tomography (CT) scans.

## 1. Introduction

Pulmonary fibrosis encompasses a collection of pulmonary ailments characterized by permanently forming scar tissue within the lung. The progressive deterioration of lung function occurs due to the stiffening and thickening of the lung interstitium, which refers to the tissues between the air sacs due to the formation of scar tissue—the circumstance mentioned above results in the demise of the individual under medical care (1). Idiopathic pulmonary fibrosis (IPF), a connective-tissue disease, and chronic pneumonitis with hypersensitivity, toxicology of drugs, and environmental exposures are commonly encountered origins of pulmonary fibrosis (2). Idiopathic pulmonary fibrosis (IPF) is frequently correlated with a poor prognosis, as observed survival durations exhibit a median range of 2–5 years (3). The histological patterns observed in pulmonary fibrosis can be classified into two primary types: usual interstitial pneumonia (UIP) and non-specific interstitial pneumonia (NSIP) (4). Idiopathic pulmonary fibrosis (IPF) is a prominent and severe manifestation of pulmonary fibrosis, distinguished by the existence of a characteristic interstitial pneumonia (UIP) structure, as observed through histological examination (5). As the disease progresses, significant fibrosis and honeycombing lead to structural changes in the lungs and impair the gas exchange (6). Patients commonly exhibit a gradual emergence of symptoms, including exertional dyspnea and dry cough, as fibrotic alterations gradually develop over months or even years. Diagnosing pulmonary fibrosis entails a thorough assessment integrating clinical, radiographic, and pathological observations. The initial step involves a comprehensive evaluation of the patient's medical background and a physical examination conducted by a respiratory physician or pulmonologist. The focus of this evaluation generally revolves around the symptoms exhibited by the patient, their medical history, and several possible danger signs that have been associated with the occurrence of pulmonary fibrosis. Various imaging modalities, including chest radiography, computed tomography (CT), and magnetic resonance imaging (MRI), are utilized to evaluate lung tissue and identify pathological abnormalities. Radiologists are pivotal in diagnosing by evaluating imaging tests and delivering a radiological diagnosis. The visual examination of computed tomography (CT) is crucial in assessing pulmonary fibrosis. Radiologists carefully examine several characteristics, including reticulation, traction bronchiectasis, and honeycombing, since these indicate this condition. A lung biopsy may be conducted to validate the diagnosis, entailing the extraction of a small specimen of lung tissue for subsequent histological analysis. Pathologists analyze lung tissue samples to establish a histopathological diagnosis, assessing various characteristics like fibrosis, inflammation, and cellular infiltration. The radiological and pathological findings demonstrate a strong correlation with the patient's clinical presentation and medical history, thereby contributing to the establishment of a conclusive diagnosis. Additional differential diagnoses, including emphysema, bronchiectasis, and interstitial lung disease, are also considered and eliminated as possibilities. Pulmonary fibrosis is effectively managed and treated by diverse healthcare experts with expertise in several disciplines. This multidisciplinary team comprises respiratory physicians, radiologists, pathologists, and surgeons. Treatment options for pulmonary fibrosis encompass a range of procedures, such as drug administration, oxygen therapy, lung transplantation, and other relevant therapeutic approaches.

Presently, there is a shortage of medical procedures that can reverse or offer a conclusive remedy for pulmonary fibrosis. Nevertheless, the utilization of pharmaceutical agents, such as nintedanib and pirfenidone, has exhibited the capacity to impede the progression of this ailment. According to sources (7, 8), it is evident that the information provided Lung transplantation is the only definitive therapeutic option for those who have reached severe stages of the disease. The prompt detection and appropriate intervention of medical disorders are paramount in preserving overall health and improving long-term outcomes. However, diagnosing this syndrome might provide challenges due to the diverse range of clinical presentations and varying advancement rates. Chest computed tomography (CT) scans are a crucial diagnostic modality for assessing pulmonary fibrosis. This technique facilitates identifying and tracking illness progression and growth over time.

The primary distinguishing feature of conventional interstitial pneumonia(UIP) is the identification of honeycombing, primarily localized in the basal and subpleural regions (9). While a medical lung biopsy is necessary to perform a conclusive diagnosis, a unique interstitial pneumonia (UIP) pattern shown on computed tomography (CT) within a pertinent clinical context is sufficient to diagnose idiopathic pulmonary fibrosis(IPF) (10). Computed tomography (CT) is crucial in analyzing medical conditions and evaluating disease severity, fibrosis distribution, and temporal progression (11). The present CT examination procedure requires a manual scan assessment by skilled radiologists. Nevertheless, this methodology is constrained by its subjective features and the possibility of differing interpretations among people (12). The utilization of quantitative CT analysis presents several benefits in terms of providing an objective and longitudinal evaluation. Nevertheless, the widespread clinical application of image processing and quantitative feature derivation needs to be improved by requiring specialized software and technical expertise (13). The domain of artificial intelligence, particularly machine learning, has experienced significant advancements, and a growing preference for the development of computer-aided diagnostic (CAD) systems.

These systems aim to automate disease classification and discover patterns in medical images. Multiple deep learning-based techniques are nowadays used to detect and classify pulmonary fibrosis. LSTM has been influential in detecting pulmonary fibrosis from chest CT scans. In contrast, Convolutional neural networks (CNNs) have been recognized as a promising methodology in the field of computer-aided diagnostic (CAD) systems to identify patterns indicative of pulmonary fibrosis through the analysis of computed tomography (CT) images (14, 15). Nevertheless, utilizing deep learning techniques necessitates a substantial quantity of meticulously curated data for training purposes, which may be laborious and financially burdensome.

Moreover, these methods can be computationally expensive and require powerful hardware. Similarly, transfer learning-based techniques like VGG, ResNet, Inception, Xception, and EfficientNet provide a higher level of accuracy because these models get pre-trained weights and fine-tune them on smaller datasets, leading to improved accuracy and faster results and less data requirement compared to training a model from scratch. However, transfer learning requires a pre-trained model relevant to the specific task, and the level of accuracy of the pre-trained model can influence the accuracy of the fine-tuned model. Another method, multimodal fusion, combines data from multiple modalities, such as medical images, clinical data, and genomic data, to detect pulmonary fibrosis. The integration of many modalities enhances the precision of pulmonary fibrosis detection. The application of this methodology is constrained due to its reliance on a substantial volume of data from many modalities, which may provide challenges in terms of acquisition. Moreover, integrating data from different modalities can be challenging, and the data quality can affect the model's accuracy.

Attention mechanisms like STN, DANet, CBAM, and ViT are deep learning models that focus on specific parts of the input data, such as particular objects or regions of an image. This can help improve pulmonary fibrosis detection accuracy by identifying the most critical features in medical images using less data and computations. Some pioneering studies have demonstrated the potential of Vision Transformer (ViT) models in medical imagery (16, 17). For example, Vision Transformer (ViT) models have shown notable precision in identifying COVID-19 by analyzing chest CT scans and X-rays (18). We have chosen to apply ViT for detection and classification in this study because of several advantages over traditional convolutional neural networks (CNNs). ViTs capture long-range dependencies in images, improving the analysis of diverse features. They are computationally efficient, handle large datasets well, and use a sparse representation that filters out irrelevant information. ViTs generalize to unseen data, are easily interpretable, and robust to image variations. They can also handle multi-modal input, such as images and text, which is helpful in medical imaging.

In lung cancer screening, ViT architectures have outperformed CNNs in classifying malignant lung nodules on low-dose CT (19). Furthermore, Vision Transformer (ViT) models have demonstrated exceptional performance in breast cancer classification using ultrasound images, surpassing existing benchmarks (20). However, CAD systems have yet to be widely adopted into clinical practice for pulmonary fibrosis. Challenges include model generalization across different scanners and protocols, integration into clinical workflows, and acceptance among radiologists (21). Standardized benchmark datasets with expert ground truth labels are lacking but are needed to evaluate model performance and clinical utility robustly. Even though deep learning holds promise for computerized pulmonary fibrosis detection, most studies have had small sample sizes and lacked external validation. Building large annotated datasets for network training is expensive and time-consuming (22). While curated open-source medical imaging datasets have catalyzed progress in domains like diabetic retinopathy and skin cancer classification (23, 24), Utilizing the OSIC Pulmonary Fibrosis Progression dataset facilitates researchers in training advanced deep learning models. The present study introduces a novel deep learning model, FibroVit, which aims to facilitate the automated identification of pulmonary fibrosis by analyzing chest computed tomography (CT) scans. The key contributions are:

1. Application of vision transformer for automated pulmonary fibrosis pattern recognition in HRCT chest.
2. Development of FibroVit, a customized Vision Transformer optimized for detecting fibrotic features and predicting pulmonary function decline.
3. Assembly of a large dataset with >5000 labeled CT scans for network training and performance benchmarking.
4. Explainability-driven validation of model predictions against expert radiologist assessments.
5. Release of source code and pre-trained models to the research community under an open-access license.

The FibroVit architecture is initialized using a vision transformer, specifically the ViT-base-patch16-224 model. The present model has undergone pre-training on the ImageNet-21k dataset, comprised of natural images. The weights from the vision transformer are utilized for this initialization process (25). Fine-tuning is then performed with lung CT scans to learn domain-specific features relevant to pulmonary fibrosis. Network training leverages the OSIC benchmark dataset. External validation is undertaken using an independent test set not used during training or hyperparameter tuning.

## 2. Related work

The introduction of Transformers in 2020 has significantly heightened the significance of the Vision Transformer model. This model relies heavily on deep learning for anomalies formalized in chest X-ray and CT images. The utilization of ViT architectures is presently growing in the domain of medical research diagnostics. A wide range of studies have extensively documented that exhibit notable accuracy (26). This paper comprehensively examines the deep learning techniques used by previous scholars in diagnosing and detecting pulmonary fibrosis disease. In the past few years, there has been a noticeable rise in scientific focus on using artificial intelligence (AI) for computer-assisted medical making decisions in the domain of pulmonary fibrosis. This attention is mainly directed toward analyzing computed tomography (CT) images. The primary reasons for this phenomenon arise from the exponential benefits that can be obtained and the inherent difficulties that arise from a clinical standpoint (27). In pathological images, scholars have proposed an algorithmic technique employing Convolutional Neural Networks (CNN) to categorize histological subclasses of lung cancer cells. Moreover, a methodology has been recommended to differentiate between benign and malignant cells (28, 29).

In their study, Shi et al. (30) researched to develop an automated method for identifying regions impacted by gastric cancer in photographs of gastric histopathology samples. The researchers employed a convolutional neural network (CNN) decoder in their methodology to extract relevant features and incorporate an attention mechanism. The samples used in this research were non-pulmonary histopathology specimens. Anthimopoulos et al. (31) utilized a deep convolutional neural network to categorize various patterns identified in lung tissue, like reticulation ground glass opacity, honeycombing, consolidation, and micronodules. The achievement was attained by meticulously examining two-dimensional patches extracted from computed tomography (CT) images. In their research, Christodoulidis et al. (32) presented a transfer learning approach that integrates multiple data sources and utilizes already trained deep convolutional neural networks. These neural networks were trained on diverse texture datasets. The primary aim of this study was to accurately classify regions of lung tissue in computed tomography (CT) scans based on their two-dimensional image characteristics. Li et al. (33) introduced a novel approach that employs an autoencoder stacking model. The model that has been suggested consists of four levels, each composed of four autoencoders. The main goal was to extract enhanced characteristics from computed tomography (CT) images. The four autoencoders were connected sequentially, forming a chain. Subsequently, the mentioned chain was linked to a densely connected layer and a softmax classifier. Consequently, the definitive model was obtained.

Amyar et al. (34) conducted a study wherein they created a deep neural network framework specifically tailored to analyze CT images. The study introduced a network design that consisted of a 10-layer encoder component and a 9-layer decoder component for picture reconstruction. An additional decoder component comprising nine layers was incorporated to accomplish picture segmentation. Xu et al. utilized a VNet and an inception residual network as their chosen methodologies for feature extraction in their inquiry. The networks were integrated with a region proposal network to effectively and accurately detect regions of interest (35). To tackle the matter of unclear boundaries in cancer images, a new approach called Boundary-Aware Transformer (BAT) is put forth by Wang et al. (36). The researchers included the boundary-wise attention gate into the Transformer design to enhance the effective exploitation of existing boundary-related knowledge. Integrating auxiliary supervision into the boundary-wise attention gate framework enables the optimization of training efficiency for the BAT model. The efficacy of their boundary-wise prior is confirmed through an experimental analysis conducted on the ISIC 2016+PH2 dataset (37) and the ISIC 2018 dataset (38).

Similarly to this, Wu et al. (39) present a feature adaptive transformer network (FAT-Net) that combines transformer branches with a convolutional neural network (CNN) in the encoder. The researchers have developed a decoder and feature adaptation module that optimizes memory usage and effectively integrates features from both branches. The initial exploration of employing a vision transformer for COVID-19 identification from computed tomography (CT) scans was conducted by Ambita et al. (40). The study used various vision transformers to complete picture classification tasks, including ViT-L 16, ViTL 32, ViT-B 16, ViT-H 14, and ViT-B 32.

In their study, Lee et al. (41) introduced a unique framework called Template Transformer Networks for Segmentation (TETRIS). This framework incorporates shape priors to enhance the segmentation process. The TETRIS technique integrates an end-to-end trainable Spatial Transformer (STN) to deform a shape template and align it with the underlying region of interest. Additionally, integrating these prior beliefs into the advanced Convolutional Neural Network (CNN) and U-Net models (42) commonly utilized for binary classification tasks, was implemented. Furthermore, a comparative analysis was conducted between U-Net and FCN by integrating the prior shape. Nevertheless, the Fully Convolutional Network (FCN) (43) exhibited suboptimal performance, resulting in a Dice Score of 0.79. Table 1 gives an outline of the results attained by earlier researchers using various models to categorize PF. We were therefore motivated to offer our optimized and enhanced deep learning model based on the prior studies and their accompanying constraints, which are listed in Table 1. This study aims to propose a model that attains the highest possible diagnostic accuracy for identifying and classifying CT scan slices that indicate the presence of pulmonary fibrosis (PF) in typical chest CT scan pictures. This model aims to reduce the amount of data needed and the duration of the training process.

**Table 1:** A comprehensive compilation of pertinent studies pertaining to the classification of PF and their associated key discoveries, benefits, and drawbacks.

**References**	**Year**	**Data Type**	**Model name**	**Key Findings**	**Advantages**	**Limitations**
Shi et al. (30)	2022	Histopathology images	CNN decoder with attention mechanism	Automated method for identifying regions impacted by gastric cancer	Attention mechanism can help to focus on relevant features	The acquisition of a substantial quantity of training samples is necessary.
Anthimopoulos et al. (31)	2016	CT images	Deep convolutional neural network	Categorized various patterns identified in lung tissue, such as reticulation, ground glass opacity, honeycombing, consolidation, and micronodules	Can extract features from 2D patches of CT images	Requires a large number of training samples
Christodoulidis et al. (32)	2017	CT images	Transfer learning with deep convolutional neural networks	Accurately classified regions of lung tissue in CT scans based on their two-dimensional image characteristics	Can be trained on a variety of texture datasets	Requires a large number of training samples
Li et al. (33)	2021	CT images	Autoencoder stacking model	Extracted enhanced characteristics from CT images	Can be trained on a small number of training samples	Can be computationally expensive
Amyar et al. (34)	2020	CT images	Deep neural network with encoder-decoder architecture	Segmented lung tissue in CT images	Can be used for both classification and segmentation tasks	Requires a large number of training samples
Xu et al. (35)	2020	CT images	VNet and inception residual network	Separated regions of interest in CT images	Can be used for both classification and segmentation tasks	Requires a large number of training samples
Wang et al. (36)	2021	Cancer images	Boundary-Aware Transformer (BAT)	Tackle the matter of unclear boundaries in cancer images	Utilizes pre-existing knowledge about boundaries	Requires a large number of training samples
Wu et al. (39)	2022	CT images	Feature adaptive transformer network (FAT-Net)	Combines transformer branches with a convolutional neural network in the encoder	Optimizes memory usage and effectively integrates features from both branches	Requires a large number of training samples

## 3. Methodology

### 3.1. System design

**Figure 1:**
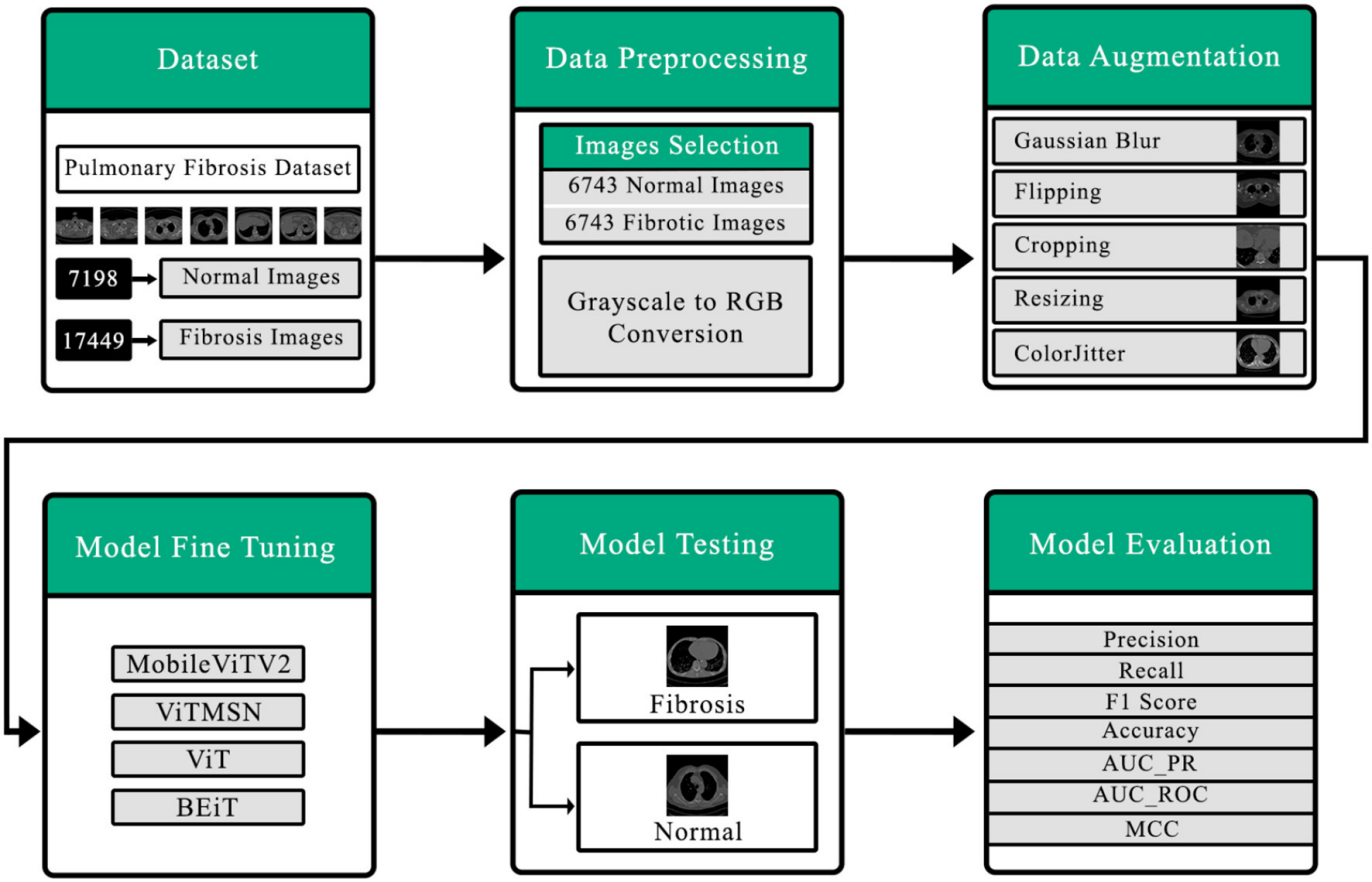
Detailed outline of the proposed system for detection of pulmonary fibrosis in chest CT scan.

Figure 1 comprehensively summarizes the present investigation. The initial stage involves the collection of the dataset, which is a crucial step. And then, there are subsequent data preprocessing procedures, such as data selection and the conversion of grayscale images into RGB. The Following stage revolves around data augmentation, aimed at rendering the data suitable for seamless integration into the vision transformer. Consequently, the model is then trained using carefully curated data. The ultimate aim of the testing model is to classify the CT scan output into normal or fibrotic categories. Ultimately, the model's performance is assessed through diverse performance metrics.

### 3.2. Dataset

**Figure 2:**
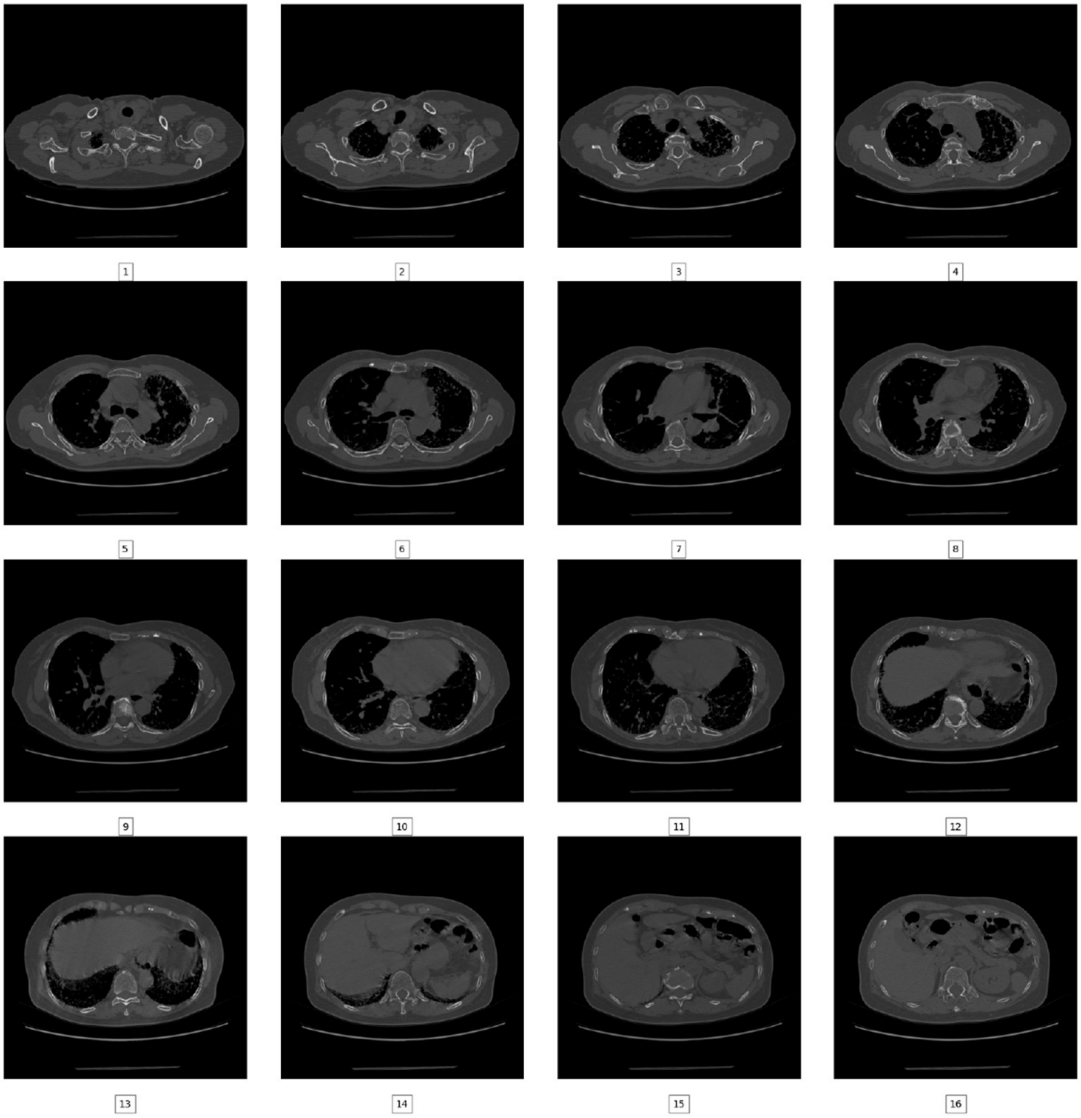
A Chest CT scan sliced into multiple images.

In this particular inquiry, the images of chest CT scans were procured from Kaggle as JPG( (PulmonaryFibrosis_dataset_Final | Kaggle). The dataset consists of images taken from a chest CT scan. There are two categories of images: normal (7,198 images) and pulmonary fibrosis (17,449 images). The data presented in Figure 2 shows the unprocessed images of each category. The chest CT scan images were partitioned into three distinct clusters, specifically the training, validation, and test sets, in accordance with an 80:10:10 ratio.

### 3.3. Data preprocessing

For detecting Fibrosis, Normal, and Fibrosis cases using ViT models, 13,486 images were selected from an initial set of 24,647 images. Of all the photos, 10,786 were assigned for training purposes, with a ratio of 80%, while 1,350 images were allocated for validation and testing purposes. The validation and testing ratios were equally set at 10 and 10%, respectively. The 80-10-10 split is a common practice in machine learning. The approach ensures an equitable data distribution for training, validation, and testing purposes. The training set, comprising 80% of the data, is used to train the model. The validation set, accounting for 10% of the data, assesses the model's performance during training. Finally, the test set, representing 10% of the data, evaluates the model's ultimate performance. A larger test set may not provide a more accurate estimate or generalize well to new data. Cross-validation provides a more robust estimate of performance. The 10% validation set can be used for cross-validation, ensuring the model generalizes well. Table 2 presents a comprehensive breakdown of the allocation of CT scan slices among distinct categories within the training, validation, and test datasets.

**Table 2:** A comprehensive categorization of chest CT scans is performed inside each class, as well as throughout the training, validation, and testing datasets.

**Dataset**	**Normal**	**Fibrosis**	**Total**
Testing	675	675	1,350
Validation	675	675	1,350
Training	5,393	5,393	10,786
Total	6,743	6,743	13,486

In the present study, performing pre-processing on the initial images obtained from CT scans of the participants in the JPG format is of utmost importance. This is necessary to ensure that the CT images are compatible with the vision transformer that has been previously established. The current study employs a range of data pre-processing methodologies, which are enumerated below

1. Randomly selecting comparable images from each category.
2. Conversion of the grayscale 2D image to an RGB image to enable its compatibility with the Vision Transformer's input format.

### 3.4. Ensuring test data separation

A strict separation between the test data and the model development process was enforced to maintain the integrity of our model's evaluation. The test dataset, consisting of 1,350 images, was kept completely isolated throughout the model training pipeline. Specifically:

- The test data was not used for model development or hyperparameter tuning.
- Only the training data (80%) and the validation data (10%) were utilized during training and fine-tuning.
- Measures were taken to prevent any accidental leakage of test data into the training or validation phases, ensuring our model's performance evaluation was conducted on unseen data.

### 3.5. Data augmentation

**Figure 3:**
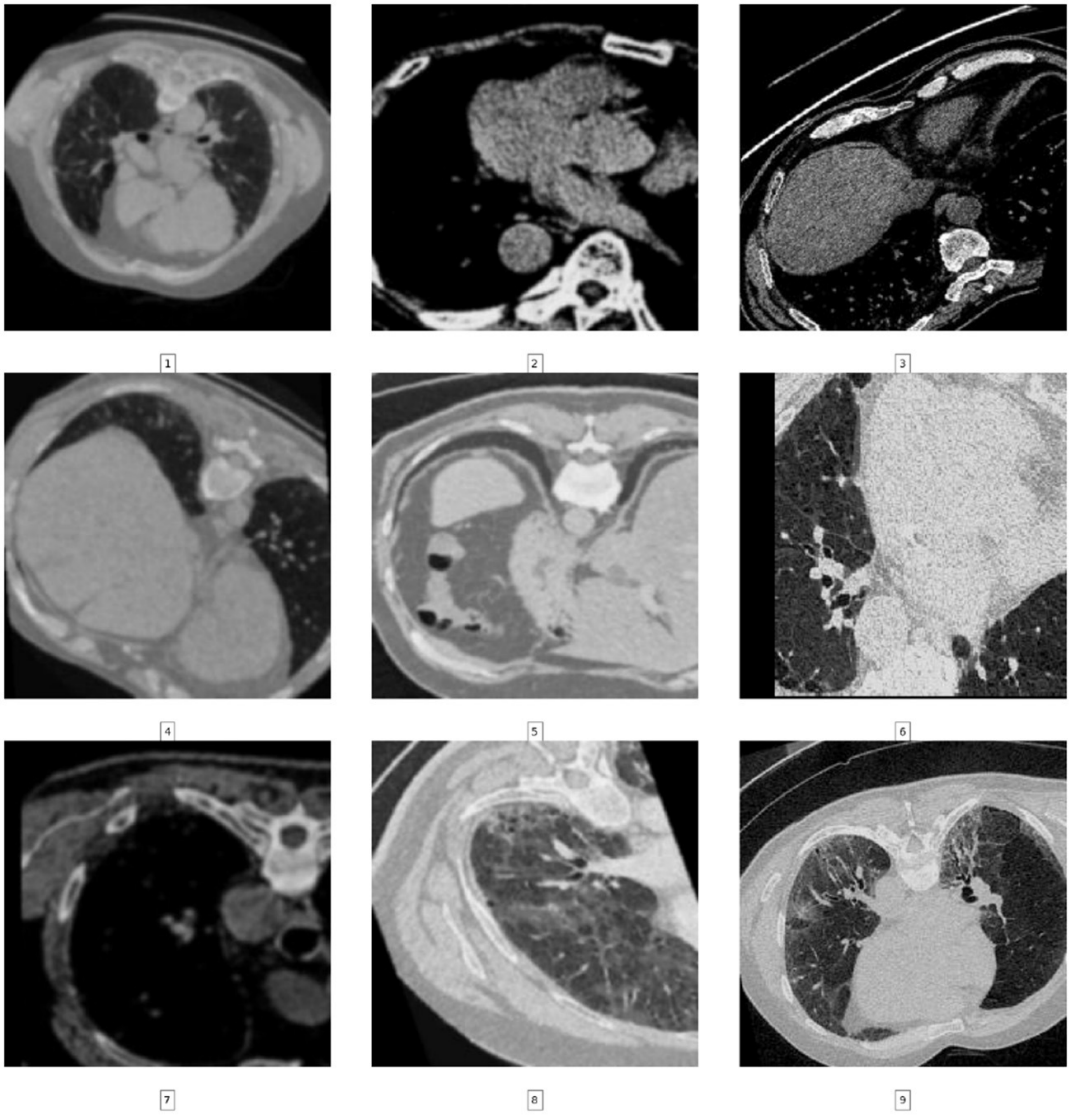
Examples of the CT images with applied augmentation techniques.

A set of geometrical augmentations, including rotation, horizontal and vertical flips, cropping, and resizing to dimensions of 224 × 224. Additionally, the image is subjected to randomized adjustments in brightness, contrast, saturation, and hue and the application of ColorJitter and Gaussian blur (Figure 3). Perceptive analytic transformations, affine transformations, affine shear transformations, and a custom lambda function with a 50% probability are also randomly applied. Moreover, the current investigation utilizes the vision transformer (vit-base-patch16-224-in21k) for binary classification, necessitating specific input image dimensions. The current models can acquire information and perform picture classification tasks by assigning class labels, namely PF and Normal. Consequently, the datasets used for training, validation, and testing were annotated by giving suitable labels to each class included within the corresponding image datasets. Furthermore, the one-hot encoding method was utilized to create a column representing the existence of the two distinct categories. A binary value of “1” was assigned to each instance in the column if the corresponding example was labeled fibrosis. In contrast, a value of “0” was assigned to normal chest CT images. A representative portion of the dataset was set aside as a test set before applying any data augmentation techniques. Data augmentation was applied exclusively to the training set, using different parameters for each image to ensure that the augmented versions of the same image had different transformations. It was ensured that test set images were not used for augmentation, as this would introduce label leakage. The original labels associated with each image were kept intact when applying data augmentation. The model's performance was evaluated exclusively on the test set after training. By doing so, it was possible to prevent overfitting to the training set and ensure that the model generalized well to unseen data.

### 3.6. Vision transformer model

**Figure 4:**
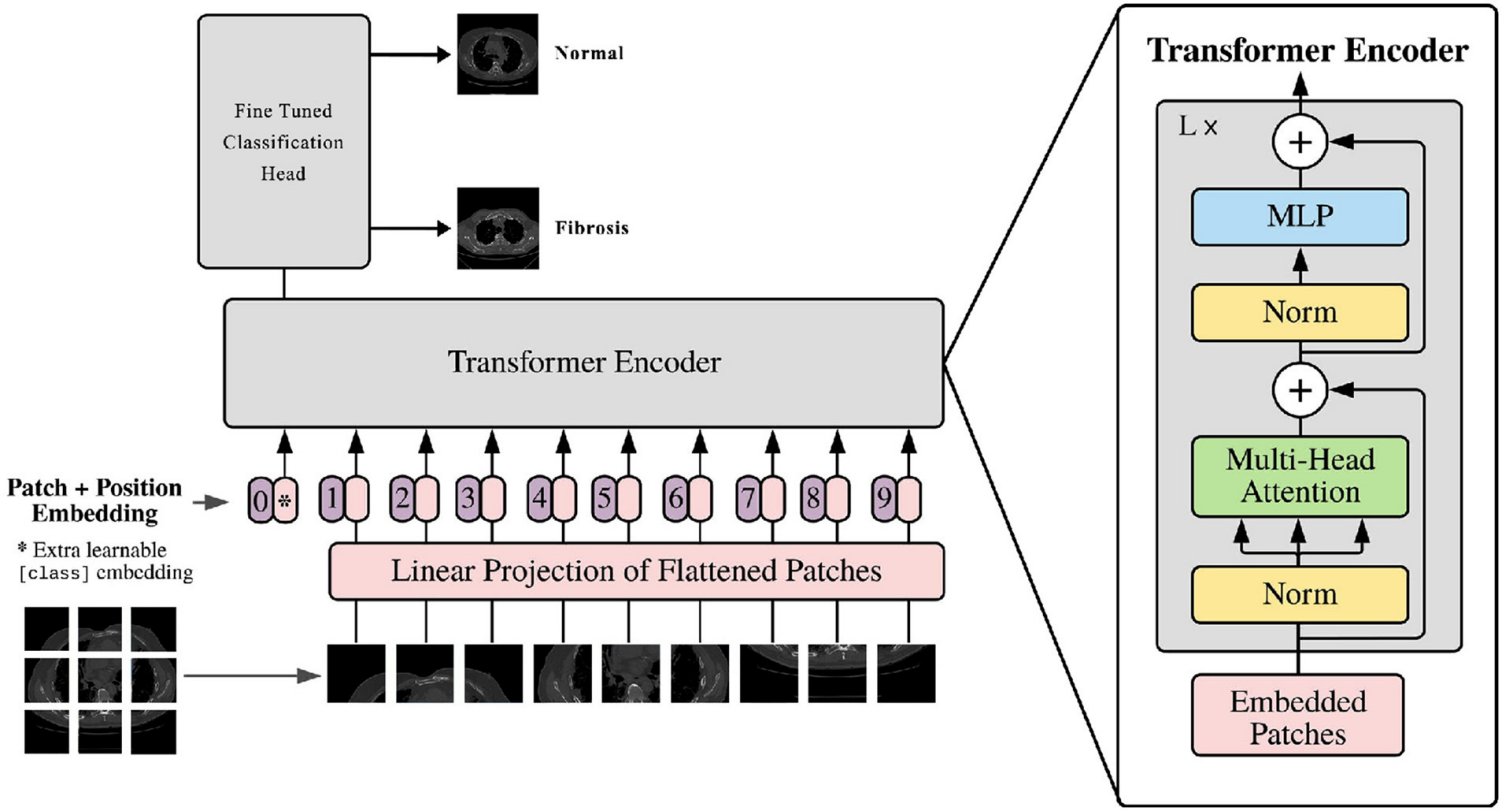
The proposed ViT model architecture for pulmonary fibrosis detection.

The vision transformer is based on the original transformer model. The input image is converted into multiple patches and fed into the model. It comprises various components, as shown in Figure 4. The vision transformer's structure is described below.

#### 3.6.1. Input image patches

The first step in the Vision Transformer (ViT) model entails dividing the input image into patches that do not overlap. The determination of the patch size adjustment is contingent upon the hyperparameter, which considers both the dimensions of the input image and the available computational resources. Subsequently, the patches are subjected to linear embedding, resulting in a sequential arrangement of vectors that the transformer may effectively process.

#### 3.6.2. Patch embedding

The patch embedding layer is like a translator that converts input patches into a sequence of numbers that a transformer model can understand. It has three parts: a linear transformation for changing the patches, a function called ReLU to make the numbers more practical, and a dropout layer. The dropout layer is a standard tool in machine learning, and it helps prevent a model from memorizing the training data too much by randomly leaving some information out. This way, the model learns to handle randomness and becomes better at making predictions.

#### 3.6.3. Positional encoding

The integration of positional encoding within the Vision Transformer (ViT) model serves the purpose of retaining the spatial information present in the input image. It improves the contextual meaning of the patch embeddings. Positional encoding is a technique that enhances each input vector within a sequence by integrating a fixed vector defined by its position. The fixed vector is achieved through training, which improves the model's understanding of the spatial connections across patches of an image.

#### 3.6.4. Encoder block

The encoder block plays an important role, in the ViT model. Its main function is to handle the sequence of patch embeddings and effectively capture their interconnections. The encoder block consists of three components; a head self-attention layer, a feed forward neural network (FFNN), and an additional multi-head self-attention layer. To determine the probabilities of the output the work, from the self-attention layer is passed through a linear layer and then subjected to a softmax activation function.

#### 3.6.5. Multi-head self-attention

The multi-head self-attention layer plays a role, in the transformer concept. It helps the model understand how different parts of the input sequence are connected. The self-attention layer measures the similarity between elements in the sequence. This information creates a weighted combination. This process is repeated times, with starting points to explore similarities. The results are then transformed linearly to produce the outcome.

#### 3.6.6. Feed-forward neural network (FFNN)

A feed-forward neural network, also known as FFNN is a type of neural network. In this network, each neuron, in one layer is connected to every neuron in the layer. This particular kind of network is mostly used in natural language processing. Its purpose is to analyze the output generated by the head self-attention layer and transform it into a feature space, with increased dimensions. The FFNN comprises two linear layers connected by a Rectified Linear Unit (ReLU) activation function. This design makes it easier for the network to combine and represent the non-linear connections between input patches.

#### 3.6.7. Classification layer

The Vision Transformer (ViT) model gives a result by taking the output from the second multi-head self-attention layer, putting it through a linear layer, and then using a softmax activation function. The softmax part of the model changes the input into a chance distribution over multiple classes so the model can give a chance distribution that includes potential paths.

### 3.7. ViT

The Vision Transformer (ViT) is a cutting-edge deep learning model that has been specifically designed for the purpose of computer vision tasks. Google Research introduced it in the year 2020 (44). It uses a transformer-based structure for computer vision tasks initially designed for natural language processing. The proposed model utilizes a decomposition technique to divide the input image into distinct patches. These patches are then transformed into a sequential arrangement of vectors by a linear embedding procedure. The patches are then subjected to multiple transformer encoder layers to capture dependencies and acquire significant image representations. The model has a classification head that makes predictions using the acquired embeddings. ViT demonstrates exceptional performance across various vision-related tasks through pre-training and fine-tuning.

### 3.8. Mobile ViT2

MobileViT2 (45) is a vision transformer model designed for efficient inference on mobile devices. It has a larger input size and more transformer encoder layers than MobileViT and utilizes regularization techniques for enhanced generalization performance. The model is pre-trained on ImageNet-21k and fine-tuned on ImageNet-1k for classification. Incorporating fused kernel additions and executed code optimizations has significantly improved inference speed, resulting in 4–5 times faster performance than the original ViT model. MobileViT2 achieves a top-1 accuracy of 75.6% on ImageNet and performs 3.2 times better than MobileViT. It represents a cutting-edge advancement in mobile vision tasks, enabling efficient and accurate performance on limited computational resources.

### 3.9. ViTMSN

ViTMSN (Masked Siamese Networks) is an architecture for self-supervised learning that aims to acquire image representations (46). The primary innovation of ViTMSN is the incorporation of a multi-scale neighborhood aggregation method, enabling the network to effectively analyze the contextual information of a picture across several scales. The objective above is accomplished by partitioning the image into patches of a predetermined size, without any overlap, and subsequently embedding these patches into a sequential arrangement of vectors using a linear approach. The subsequent sequence is inputted into a transformer encoder, which is processed by self-attention mechanisms to acquire knowledge about the interconnections among various image components. ViTMSN has been shown to achieve state-of-the-art performance on several image classification benchmarks, including ImageNet. Its ability to capture long-range dependencies and contextual information has made it a popular choice for object detection, segmentation, and generation tasks.

### 3.10. BEiT

The BEiT (47) model is a vision transformer model for image representation learning. It uses a bidirectional transformer encoder architecture to learn contextual representations of image patches.The primary concept is pre-training the model by predicting masked visual tokens based on contextual information, like BERT's masked language modeling technique. This enables the model to acquire significant visual representations without depending on manual image annotations. Fine-tuning the pre-trained BEiT model on various downstream tasks, such as image classification, has demonstrated exceptional performance, leading to state-of-the-art outcomes. In general, the findings of the BEiT study show that bidirectional transformers can acquire very effective representations from visual input through self-supervised learning, thereby diminishing the reliance on annotated image datasets.

## 4. Algorithm

1. Initialize Hyperparameters:
learning_rate = 0.0001
batch_size = 56
num_epochs = 1
weight_decay_rate = 1.0
2. Create Base Model:
base_model = ViT_Base_Patch16_224
3. Build Fibrosis Model:
fibrosis_model = base_model.output
fibrosis_model = GlobalAverage
Pooling2D
if dropout_rate > 0: fibrosis_model =
Dropout(0.5)(fibrosis_model)
( if dropout_rate > 0)
fibrosis_model = Dense(len(classes)
,activation="sigmoid")
(fibrosis_model)
4. Build the Complete Model:
model = Model(inputs=base_model. input,
outputs=fibrosis_model)
5. Set Base Model Layers as Trainable:
for layer in base_model.layers:
layer.trainable = True
6. Create Callbacks:
callback_list = [... ]
7. Model Compilation:
model.compile(loss="binary
_crossentropy",optimizer="adam",
metrics=["accuracy"])
8. Train the Model:
history = model.fit_generator
(train_generator, epochs=num_epochs,
validation_data=val_generator,
callbacks=callback_list)

## 5. Model training

For detecting Fibrosis, Normal, and Fibrosis cases using ViT models, 13,486 images were selected from an initial set of 24,647 images. Of all the photos, 10,786 were assigned for training purposes, with a ratio of 80%, while 1,350 images were allocated for validation and testing purposes. The validation and testing ratios were equally set at 10 and 10%, respectively. The application of the data augmentation approach was used in order to enhance the overall quality of the training process and validation data. Furthermore, the models underwent a process of fine-tuning for a single epoch, employing an Adam optimizer alongside a consistent learning rate of 0.0001. The ViT-Base-Patch16-224 model was configured with a batch size of 56. It is important to mention that the optimal hyperparameters were determined using experimental methodologies. In the evaluation context, various metrics were utilized, encompassing Precision, Recall, F1 Score, Accuracy, AUC_PR, AUC_ROC, and Matthews Correlation Coefficient (MCC). The algorithm's pre-processing, development, and evaluation were done using Python and Pytorch on a powerful NVIDIA Tesla T4 GPU and 12 GB of RAM.

## 6. Model optimization

The optimization process for the model involved the systematic tuning of two critical hyperparameters: batch size and learning rate. Initially, a conservative batch size of 4 was selected. However, larger batch sizes were considered and evaluated as the optimization progressed. Ultimately, it was determined that a batch size of 56 was the most promising choice, providing a balance between computational efficiency and model convergence. Simultaneously, the learning rate, which governs the step size taken during gradient descent, was subjected to meticulous adjustments. The learning rate was refined through iterative experimentation, starting from an initial value 0.01. After a thorough exploration, a substantially reduced learning rate of 0.0001 was identified as the optimal choice for the model, exhibiting the most consistent performance improvements. The model's accuracy and generalization exhibited remarkable enhancements by optimizing these two fundamental parameters, surpassing its earlier iterations. This meticulous optimization process ultimately enabled the model to achieve superior results in its intended tasks while efficiently utilizing computational resources. The optimization of the model's fundamental parameters was a long and an iterative process that required carefully balancing performance gains with computational costs. Many rounds of experiments were conducted to tune the hyperparameters governing model capacity and regularization. The final optimized model demonstrated significantly improved accuracy and generalization ability compared to prior versions.

## 7. Performance evaluation metric

To conduct a comprehensive performance evaluation, the pre-trained vision transformer underwent an assessment on independent 10% test data using a range of parameters such as Recall, F1 Score, Accuracy, Precision, AUC_PR, AUC_ROC, and MCC.


$$\begin{array}{c} Accuracy=\frac{TP+TN}{TP+TN+FP+FN}\\ Precision=\frac{TP}{TP+FP}\\ Recall=\frac{TP}{TP+FN} \end{array}$$

$$\begin{array}{c} F1=\frac{2\cdot Precision\cdot Recall}{Precision+Recall}\\ Sensitivity=\frac{TP}{TP+FN}\\ Specificity=\frac{TN}{TN+FP} \end{array}$$

The process of categorizing patient CT scan slices into two distinct groups, specifically pulmonary fibrosis (PF) and normal CT scans, involved the application of specific terminology, including true negative (TN), true positive (TP), false negative (FN), and false positive (FP). The terminology mentioned above was employed to denote the precise recognition of PF-positive pictures (true positives or TP), the exact identification of standard CT images (true negatives or TN), the erroneous classification of classic CT images as PF-positive (false positives or FP), and the failure to recognize PF-positive images (false negatives or FN), correspondingly. The examination of the suggested technique was carried out by employing a combination of image-based and case-based evaluations. The assessment of IPF probability was conducted using image-based evaluation, wherein patch images were presented to the Vision Transformer (ViT) model, and the resulting outcomes were gathered. The average likelihoods of ViT-output IPF were computed for each instance in the case-based classification. Subsequently, this value was employed to differentiate instances of idiopathic pulmonary fibrosis (IPF) from those that were not indicative of IPF.

## 8. Results

**Figure 5:**
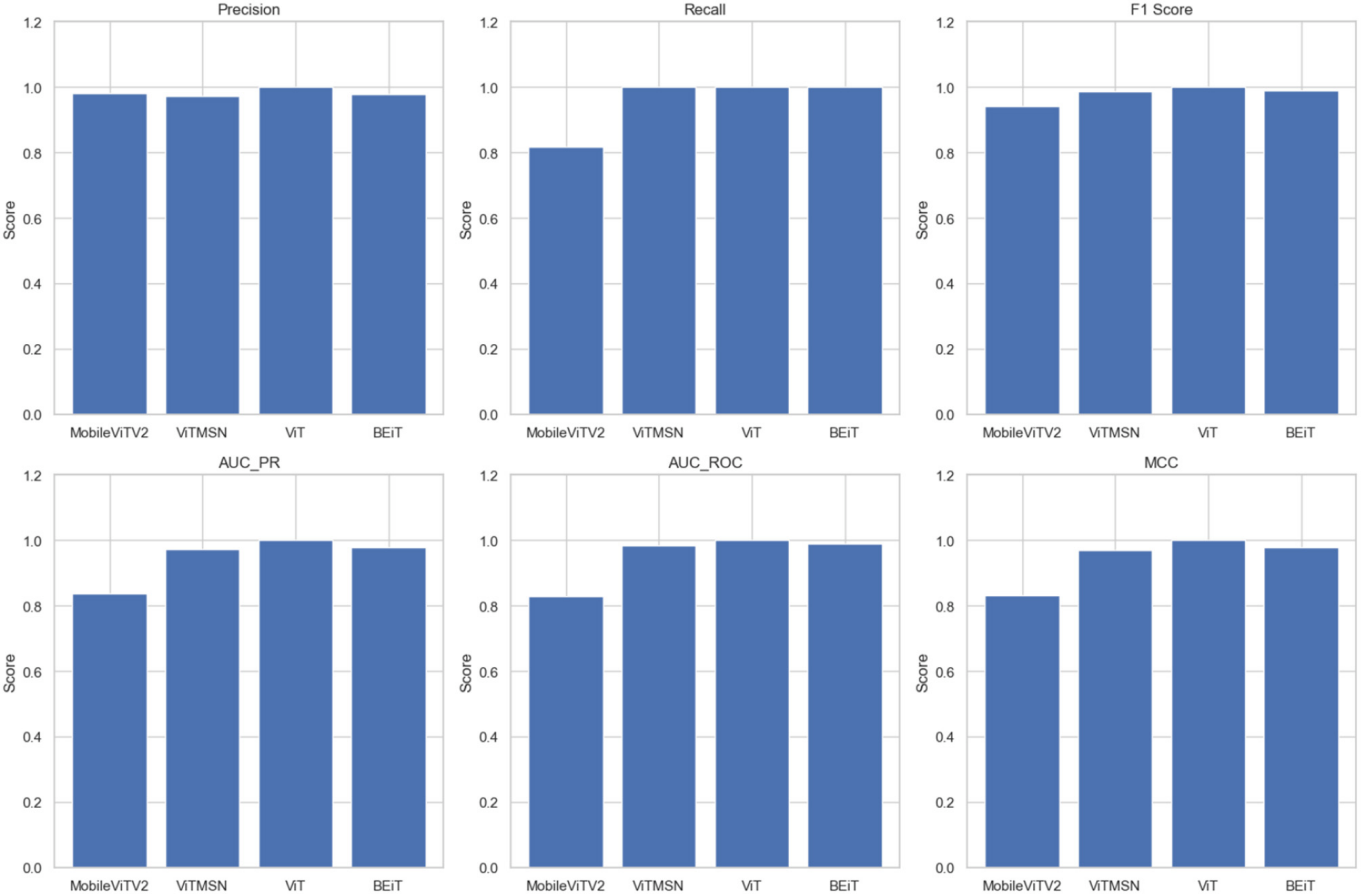
The values of Precision, Recall, F1 Score, AUC PR, AUC ROC, and MCC of four vision transformers.

The present study involved fine-tuning and validating already present Vision Transformer models to accurately categorize slice images of chest CT into Normal and PF case. The performance of four sophisticated Vision Transformer models, MobileViTV2, ViTMSN, ViT, and BEiT, was assessed using established performance measures. The models were optimized using the AdamW optimizer, employing a constant learning rate of 0.0001. Regarding validation accuracy, MobileViTV2 obtained a commendable accuracy rate of 98.36%, but ViTMSN beat it with an even higher accuracy rate of 99.63%. The ViT model demonstrated exceptional performance, attaining a validation accuracy of 99.85%, and BEiT also performed well with a validation accuracy of 99.78%. Moving on to testing accuracy, MobileViTV2 recorded a testing accuracy of 90.29%. ViTMSN showed robustness with a testing accuracy of 98.51%. Remarkably, the ViT model achieved a perfect testing accuracy of 100%, showcasing its remarkable ability to classify chest CT slice images. MobileViTV2 had a training loss of 1.8041, ViTMSN exhibited a significantly lower training loss of 0.2722, while the ViT model demonstrated remarkable efficiency with a training loss of just 0.0024. BEiT also performed well, with a training loss of 0.0269. Validation loss, another critical metric, demonstrated the ViT model's excellence again, with a shallow value of 0.0054. MobileViTV2 had a validation loss of 0.1391, ViTMSN of 0.0191, and BEiT of 0.0082, all indicating their capability to maintain low error rates during validation. The performance evaluation of selected models was performed using Precision, Recall, F1 Score, AUC_PR, AUC_ROC, and MCC. Precision, which measures the accuracy of positive predictions, was exceptional for ViT, standing at a perfect 1.0. The other models, MobileViTV2, ViTMSN, and BEiT, also showed high precision values of 0.9823, 0.9712, and 0.9782, respectively, highlighting their ability to make accurate positive predictions. Recall, representing the proportion of actual positives correctly predicted, was perfect (1.0) for ViT, ViTMSN, and BEiT, indicating their capability to capture all positive cases. MobileViTV2 had a slightly lower recall of 0.8177. The F1 Score, which balances precision and recall, reached a perfect 1.0 for ViT, while the other models were close, with values of 0.9424 for MobileViTV2, 0.9854 for ViTMSN, and 0.9890 for BEiT. AUC_PR (Area Under the Precision-Recall Curve) was robust for all models, with MobileViTV2 having the lowest value at 0.8366. ViT MSN has a value of 0.9712, and BEiT has 0.9782. The ViT again outperformed other models with value 1. AUC_ROC (Area Under the Receiver Operating Characteristic Curve) also showed strong performance, with MobileViTV2 having the lowest value at 0.8276 and ViT with the outstanding 1. ViTMSN has an AUC_ROC of 0.9851 BEiT of 0.9888. Lastly, the Matthews Correlation Coefficient (MCC) assessed binary classification quality, and all models exhibited high MCC values, with MobileViTV2 having the lowest at 0.8312 while ViTMSN 0.9707, BEiT 0.9780 and ViT with 1, emphasizing the effectiveness of ViT model in classifying chest CT slice images into Normal and PF cases. The performance of the four Vision Transformer models, ViT, ViTMSN, BEiT, and MobileViTV2, in categorizing chest CT slice images into Normal and PF cases, was evaluated using standard performance metrics. ViT achieved the highest sensitivity and specificity, with values of 1 and 1, respectively. ViTMSN had a sensitivity of 1.0 and a specificity of 0.9837, while BeIT had a sensitivity of 0.9955 and a specificity of 1.0. MobileViTV2 had a sensitivity of 1.0 and a specificity of 0.9779. These results suggest that all four models performed well in accurately categorizing chest CT slice images, with ViT and BeIT being the top performers. The results obtained from utilizing a distribution of 80% training data, 10% validation data, and 10% testing data to fixed optimized learning rate for four vision transformer learning models over the course of an epoch can be found in Table 3 and Figure 5. The optimal learning rate was achieved through a sequence of iterative procedures.

**Table 3:** A comparative assessment of the performance exhibited by four vision transformer models under a constant learning rate.

**Model**	**Optimizer**	**Learning rate**	**Validation accuracy (%)**	**Testing accuracy (%)**	**Training loss**	**Validation loss**
MobileViTV2	AdamW	0.0001	98.36	90.29	1.8041	0.1391
ViTMSN	AdamW	0.0001	99.63	98.51	0.2722	0.0191
ViT	AdamW	0.0001	99.85	100	0.0024	0.0054
BEiT	AdamW	0.0001	99.78	98.88	0.0269	0.0082

**Figure 6:**
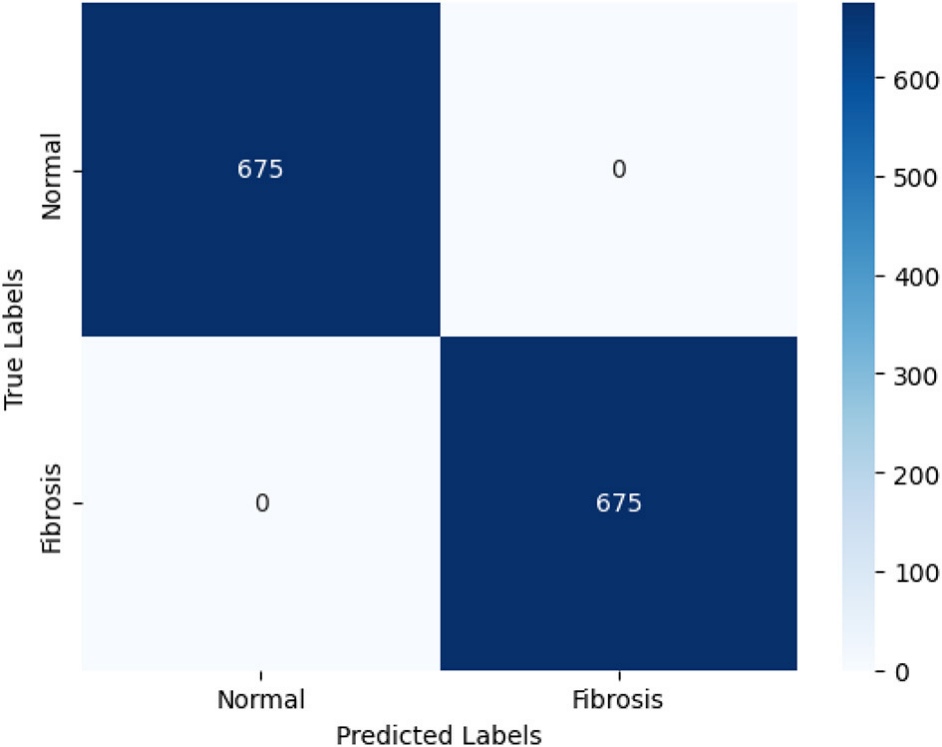
The confusion matrix depicting the performance of the highest-performing Vision Transformer (ViT) model is presented below. The ViT model can accurately categorize 675 out of 675 photos according to the confusion matrix. The 675 correctly identified photos are divided into two classes: 675 are in the positive (PF) class and 675 are in the negative (normal) class.

The Vision Transformer (ViT) model, trained with a learning rate of 0.0001, demonstrated exceptional performance in accurately categorizing Fibrosis positive images. The model attained a level of accuracy for validation of 99.85% and an accuracy in the testing of 100%. Furthermore, the model exhibited validation and training losses of 0.047 and 0.075 correspondingly. The capability of ViT to identify pulmonary fibrosis in its initial stages is particularly remarkable. The model demonstrated exceptional performance with a testing accuracy of 100%, suggesting its proficiency in detecting cases during their early stages. The confusion matrix shown in Figure 6 describes its accuracy. Early detection can enhance patient outcomes and facilitate more efficient treatment planning. The implementation of timely interventions has the potential to decelerate the advancement of the disease, mitigate symptoms, and improve the prognosis. In contrast, the ViT model demonstrates a remarkable equilibrium between accuracy and recall, achieving a precision and recall value of 1.0, thereby reducing false positives and false negatives. The degree above of precision guarantees timely medical care to people in need while concurrently minimizing superfluous examinations or procedures. The high precision and recall of ViT contribute to enhanced diagnostic accuracy, hence mitigating the impact of false alarms.

The excellent diagnostic accuracy of ViT results in improved resource allocation efficiency within healthcare settings. By reducing the number of false-positive cases, there is a decrease in the need for unneeded diagnostic procedures, resulting in cost savings and improved utilization of resources. This intervention has advantageous implications for healthcare practitioners as it enhances the overall patient experience by reducing unneeded interventions. The capacity of ViT to promptly detect and manage instances of pulmonary fibrosis holds considerable ramifications for public health. The implementation of timely intervention, supported by precise diagnosis, has the potential to mitigate the total societal impact of the condition. This phenomenon can lead to reduced healthcare expenditures, a drop in hospital admissions, and enhanced long-term management of pulmonary fibrosis on a broader scope, significantly contributing to strengthening public health.

The Vision Transformer (ViT) model demonstrates superior performance to other vision transformer models in the specific classification task, attaining a testing accuracy of 100%. The model achieves perfect recall and precision, with a rate of 100%, when categorizing PF and regular chest CT scan images. The Vision Transformer (ViT) model demonstrates a high level of suitability in accurately categorizing pulmonary fibrosis (PF) images when compared to standard chest computed tomography (CT) images, as indicated by a perfect F1-score, Area Under the Curve (AUC) and Matthews Correlation Coefficient (MCC) of 1.00. The already trained Vision Transformer models achieve peak accuracy within a single epoch. Based on the data presented in Table 4, it is evident that the already-trained Vision Transformer (ViT) model exhibits superior performance compared to the other pre-trained deep learning models investigated in the research. The ViT, which has been pre-trained, demonstrates proficient classification capabilities when applied to PF-positive images. The Vision Transformer (ViT) model demonstrates enhanced precision, accuracy, Matthews correlation coefficient (MCC), AUC_PR and ROC_AUC, and F1-score.

**Table 4:** A comparison of different performance metrics between the four vision transformers.

**Model**	**Precision**	**Recall**	**F1 Score**	**AUC_PR**	**AUC_ROC**	**MCC**	**Sensitivity**	**Specificity**
MobileViTV2	0.9823	0.8177	0.9424	0.8366	0.8276	0.8312	1	0.9779
ViTMSN	0.9712	1	0.9854	0.9712	0.9851	0.9707	1	0.9837
ViT	1	1	1	1	1	1	1	1
BEiT	0.9782	1	0.9890	0.9782	0.9888	0.9780	0.9955	1

## 9. Discussion

Pulmonary fibrosis is a medical illness that poses a possible threat to life and is distinguished by the absence of a conclusive remedy. Therefore, it is crucial to diagnose the condition quickly and efficiently. The scientific community has proposed a variety of image processing and deep learning models as potential solutions to tackle this problem.

The present investigation introduces an optimized, fine-tuned pre-existing vision transformer model to differentiate between pulmonary and non-pulmonary fibrosis CT scans. Initially, the CT scan images were preprocessed to meet the prerequisites of the vision transformer. It was pursued by executing data augmentation methods such as rotations, flips, cropping, resizing, and randomized adjustments to brightness, contrast, saturation, and hue. Moreover, other techniques, including ColorJitter, Gaussian blur, affine transformations, and a custom lambda function, were randomly employed during training to avert overfitting.

Subsequently, the models underwent fine-tuning with hyperparameters, such as epochs and learning rate, to enhance their performance for the classification task. The four Vision Transformer models, MobileViTV2, ViTMSN, ViT, and BeiT, were used to select the best model. The fine-tuned ViT model exhibited the highest validation (99.5%) and testing accuracy (100%) using CT images.

The Vision Transformer (ViT) model also demonstrated a minimal loss function of 0.0075 during the training phase and 0.0047 during the validation phase, despite training for a single epoch. Furthermore, during the assessment of the ViT on the independent test data subset, which accounted for 10% of the entire dataset, it demonstrated outstanding performance across a range of evaluation metrics. The measures employed in this study encompassed the precision-recall curve, accuracy, F1-score, precision, the area under the precision-recall curve (AUC_PR), area under the receiver operating characteristic curve (ROC_AUC), Matthew's Correlation Coefficient (MCC), as well as the Sensitivity and Specificity score, both of which were observed to be 1.00.

The MobileLungNetV2 system, as stated in (48), demonstrated a high accuracy rate of 96.97% in classifying lung texture or patterns associated with lung diseases, utilizing X-ray images. The PGGAN model introduced in (49) exhibited a detection sensitivity of 97.2% for classification performance. In (50) where different deep learning, pre-trained CNN models were used to classify pulmonary fibrosis, the modified, pre-trained ResNetv50V2 model had a 100% accuracy rate.

Our proposed pre-trained ViT model exhibits the highest test accuracy of 100%, validation accuracy of 99.85%, training loss of 0.0075, and validation loss of 0.0047. The remarkable achievement was attained within a solitary training epoch, surpassing the efficiency of all alternative deep learning techniques models.

The current study makes notable advancements in identifying pulmonary fibrosis using deep-learning models. In terms of precision, effectiveness, and resilience, the ViT model being offered demonstrates superior performance compared to other cutting-edge models, namely MobileLungNetV2, PGGAN, and modified ResNetv50V2. The vision transformer model, first designed to classify natural images, has demonstrated potential in medical image analysis, particularly in the accurate diagnosis of pulmonary fibrosis. The results of this work highlight the importance of employing transfer learning and data augmentation methods to enhance the efficacy of deep learning models in the context of medical picture classification tasks. The findings of this study are of significant relevance in the clinical context, as the precise and prompt identification of pulmonary fibrosis plays a crucial role in impacting patient outcomes. The excellent accuracy and efficiency demonstrated by the ViT model indicate its potential use as a diagnostic tool for pulmonary fibrosis among radiologists and clinicians. This might lead to a reduction in the time and resources now expended in the diagnostic process. Subsequent investigations may delve into incorporating the Vision Transformer (ViT) model within clinical processes to assess its efficacy within authentic operational contexts.

A multidimensional approach is essential to validate and seamlessly integrate the Vision Transformer (ViT) model into clinical practice. This includes external validation on diverse datasets to ensure the model's generalizability, clinical validation through real-world testing with the involvement of healthcare professionals, and a keen focus on ethical and legal aspects such as patient consent and data privacy. Moreover, efforts should be directed toward improving model interpretability to make its decisions more transparent and understandable for clinicians. Continuous monitoring and collaboration with radiologists and clinicians play a vital role in enhancing the performance of the model and tackling practical obstacles. Clear clinical guidelines and standards should be established, outlining when and how the model should be used, and cost-effectiveness should be assessed to determine the impact on patient care. By meticulously addressing these considerations, the ViT model can become a valuable diagnostic tool, enhancing the accuracy and efficiency of pulmonary fibrosis diagnosis in clinical settings.

## 10. Conclusions and future work

This study showcases the enhanced performance of the Vision Transformer in accurately detecting and differentiating pulmonary fibrosis from chest CT scans. Our methodology has attained classification accuracy at the cutting edge of current research in the field, specifically in the classification of CT images exhibiting positive pulmonary fibrosis (PF) characteristics. This achievement surpasses the performance of similar approaches documented in recent literature.

**Figure 7:**
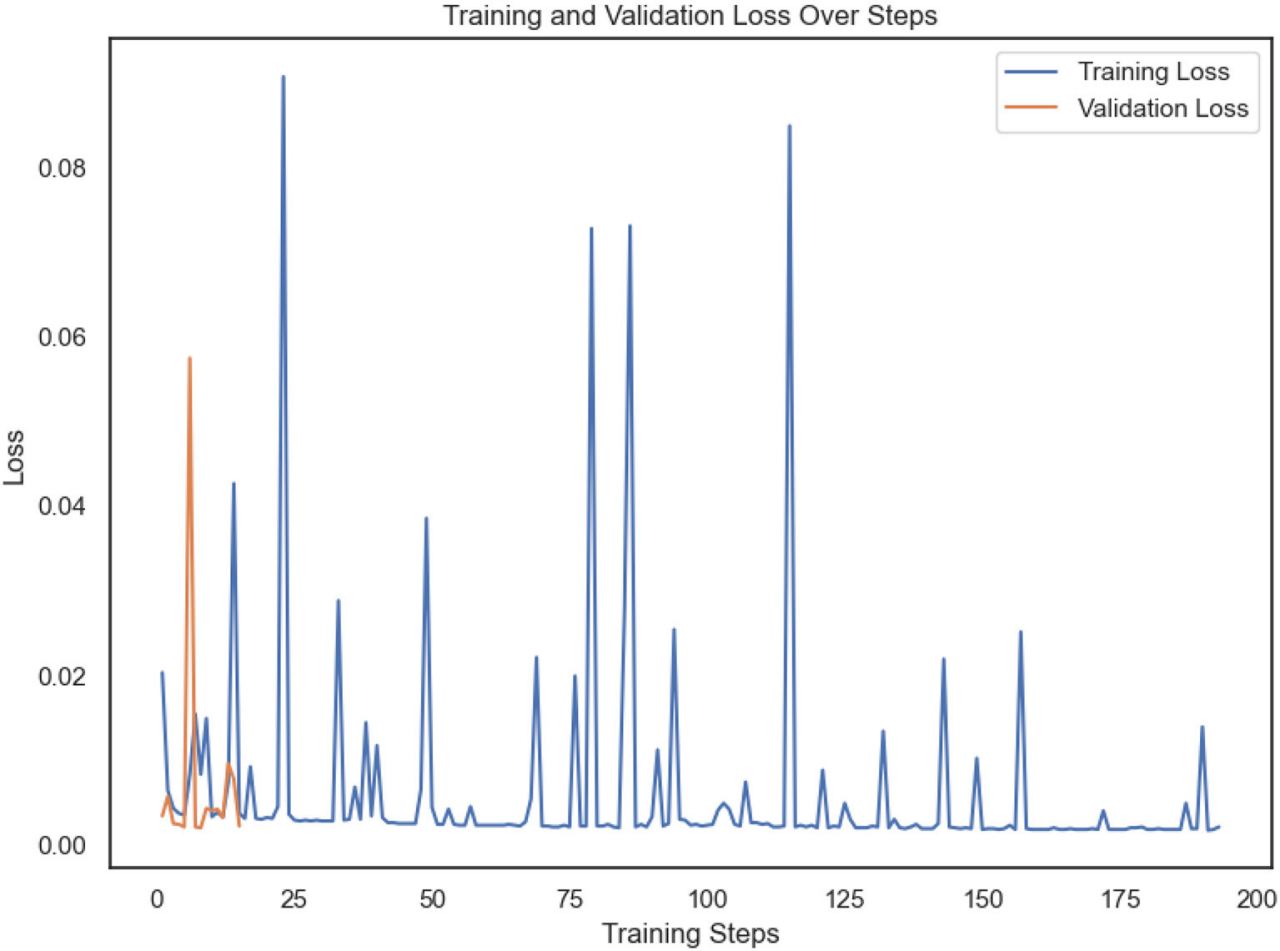
The training loss of one epoch with 193 steps and validation loss of 15 steps.

After undergoing optimization, the Vision Transformer (ViT) model exhibited outstanding performance in terms of classification accuracy. The model demonstrated accuracy for validation of 99.85% and attained a flawless accuracy of 100% when utilizing data obtained from a chest CT scan. The Vision Transformer (ViT) achieved a training dataset loss equal to 0.0024 and a validation dataset loss of 0.0054 following a single training session single training session as shown in Figure 7.

Future objectives include optimizing the ViT elements through ablation studies and expanding the dataset to handle multi-class classification of various interstitial lung diseases. The model will be trained to differentiate between diseases including emphysema, bronchiectasis, and sarcoidosis with high accuracy. Additionally, transfer learning and ensemble learning techniques will be explored to enhance performance. The ViT models demonstrate a notable level of precision and can autonomously detect and classify diverse respiratory ailments, encompassing COVID-19, pneumonia, and lung cancer. Evaluating the trained model's precision in detecting specific regions of interest in CT scan pictures illustrating pulmonary fibrosis requires a crucial collaborative alliance with medical professionals. This study's main aim is to evaluate the healthcare practitioner model's correctness comprehensively. To optimize the performance of the Vision Transformer (ViT) model, our study aims to conduct ablation experiments to assess the influence of several components, including the loss function, optimizer, flatten layer, learning rate, and pre-trained models, on its overall accuracy. This approach aims to augment the resilience of designs to boost their efficacy in addressing classification tasks. To enhance the generalization capabilities of ViT across diverse patient groups and image variations, our study aims to explore the application of modern data augmentation approaches. Furthermore, our attention will be directed toward enhancing the comprehensibility of the model's prognostications by generating heatmaps that accentuate regions of significance within CT scans. A comprehensive clinical validation process will assess the model's practicality in real-world scenarios. This process will involve blind testing using diverse CT scans obtained from multiple medical centers and the participation of healthcare specialists.

We will explore the possibility of integrating other medical imaging modalities, such as magnetic resonance imaging (MRI) or positron emission tomography (PET) scans, to expand the model's diagnostic capabilities like detection of the specific types of fibrosis patterns. Transfer learning methods will be investigated to adapt the model for detecting other lung diseases, broadening its clinical utility. We will also assess the feasibility of deploying the model in healthcare settings, including computational requirements, scalability, and regulatory compliance. To ensure responsible and secure deployment in healthcare environments, we will address ethical considerations such as data privacy and regulatory compliance (e.g., HIPAA or GDPR). By following these comprehensive steps, we can advance the application of the Vision Transformer in diagnosing pulmonary fibrosis and other lung diseases, ultimately improving patient care and healthcare outcomes.

Our objective is to optimize the performance of the Vision Transformer in diagnosing lung diseases by conducting ablation studies, improving its generalization capabilities, interpretability, and clinical utility, and addressing ethical considerations. By following a structured approach, we can ensure the real-world applicability and effectiveness of this powerful tool in improving patient care and healthcare outcomes.

## Data availability statement

Publicly available datasets were analyzed in this study. This data can be found at: https://www.kaggle.com/datasets/icmicm/pulmonaryfibrosis-dataset-final/code?datasetId=1411520&amp;sortBy=relevance.

## Ethics statement

Ethical review and approval was not required for the study on human participants in accordance with the local legislation and institutional requirements. Written informed consent from the patients/ participants was not required to participate in this study in accordance with the national legislation and the institutional requirements.

## Author contributions

MW: Conceptualization, Methodology, Visualization, Writing—original draft. MF: Investigation, Methodology, Supervision, Writing—review & editing. NA: Formal analysis, Funding acquisition, Project administration, Resources, Writing—review & editing. MA: Investigation, Resources, Validation, Writing—review & editing, Funding acquisition. GS: Formal analysis, Investigation, Methodology, Validation, Writing—review & editing.
